# Development and validation of a predictive model for perioperative low-density lipoprotein as a risk factor for postoperative cerebral infarction in Moyamoya disease

**DOI:** 10.3389/fneur.2025.1602006

**Published:** 2025-05-22

**Authors:** Jinpeng Wu, Yifan Xu, Chonghui Zhang, Cuiping Mu, Le Yu, Haowen Xu, Chao Wang, Zhenwen Cui

**Affiliations:** ^1^Department of Neurosurgery, The Affiliated Hospital of Qingdao University, Qingdao, China; ^2^Department of Nephrology, Qingdao Municipal Hospital, Qingdao, China; ^3^Department of Vascular surgery, The Affiliated Hospital of Qingdao University, Qingdao, China

**Keywords:** Moyamoya disease, postoperative cerebral infarction, low-density lipoprotein, prediction model, dynamic monitoring

## Abstract

**Background:**

Moyamoya disease (MMD) is a rare progressive cerebrovascular disorder with a high risk of postoperative cerebral infarction. Low-density lipoprotein (LDL) is a key risk factor for atherosclerosis, but the association between perioperative dynamic changes in LDL levels and the risk of postoperative cerebral infarction in MMD patients has not been thoroughly studied.

**Methods:**

This retrospective, single-center study included 266 MMD patients who underwent surgical treatment at The Affiliated Hospital of Qingdao University between 2015 and 2022. Preoperative, 24-h postoperative, and recovery-phase LDL levels (minimum, maximum, and mean) were recorded. Key variables were selected using LASSO regression, and a risk prediction model for cerebral infarction was constructed using multivariate logistic regression analysis.

**Results:**

Among the 266 patients, preoperative LDL (*p* = 0.049), postoperative LDL (*p* = 0.027), and mean LDL during the recovery period (*p* = 0.036) were significantly associated with the occurrence of postoperative cerebral infarction. The integrated model, combining LDL indicators and clinical variables, demonstrated excellent predictive ability (AUC = 0.82) and good calibration. Decision curve analysis (DCA) further validated the model’s application in clinical decision-making, indicating its effectiveness in identifying high-risk patients.

**Conclusion:**

Dynamic monitoring of LDL levels during the perioperative period is of great significance for predicting the risk of postoperative cerebral infarction in MMD patients. The constructed risk prediction model provides a scientific basis for early identification of high-risk patients and the development of individualized intervention strategies, with the potential to improve clinical management and patient outcomes.

## 1 Introduction

MMD is a rare, progressive cerebrovascular disorder characterized by the progressive stenosis of the terminal portion of the internal carotid artery and its compensatory collateral capillary branches. This leads to the formation of compensatory vessels at the base of the brain, which can result in severe neurological deficits and even death (1, 2). Despite significant advancements in understanding the pathophysiology, diagnosis, and treatment of MMD in recent years, perioperative complications, particularly the incidence of postoperative cerebral infarction, remain high, presenting a major challenge for clinical management (3, 4). Current research primarily focuses on the pathological mechanisms, imaging features, and surgical treatment of MMD, while perioperative risk assessment and management remain underexplored (5).

LDL, a key risk factor for atherosclerosis, has gained increasing attention among cardiovascular and cerebrovascular researchers in recent years (6, 7). LDL is not only closely associated with cerebrovascular diseases but may also play an important role in the pathophysiology of MMD. However, most existing studies have focused on assessing LDL levels at a single time point (8, 9), neglecting the impact of dynamic LDL fluctuations on perioperative cerebral infarction risk. With the continuous development of imaging technologies, molecular biology methods, and statistical techniques, dynamic monitoring and analysis of multi-timepoint data have become critical tools for elucidating the pathogenesis of complex diseases (10). For MMD patients, changes in LDL levels during the perioperative period may reflect overall metabolic state and endothelial function fluctuations, which in turn may influence the risk of postoperative cerebral infarction (11). Therefore, investigating the dynamic changes in LDL levels during the perioperative period and their predictive value for postoperative cerebral infarction risk holds significant clinical and academic importance.

Postoperative cerebral infarction refers to an ischemic stroke that occurs after surgery due to reduced blood flow to the brain, resulting in tissue damage. In MMD patients, fluctuations in LDL levels during the perioperative period may reflect changes in metabolic and endothelial function, influencing the risk of infarction (12).

Several prospective and retrospective studies have shown that LDL levels in MMD patients fluctuate significantly before and after surgery, and these fluctuations are closely linked to the occurrence of postoperative adverse events, including cerebral infarction (13–15). However, current research remains limited by a lack of large-scale, systematic, and multi-indicator analyses. The relationship between dynamic LDL fluctuations and postoperative cerebral infarction risk has yet to be comprehensively explored. Thus, this study aims to monitor dynamic changes in LDL levels during the perioperative period in MMD patients, integrate multiple clinical indicators, and construct a predictive model to assess the risk of postoperative cerebral infarction. By incorporating key LDL indicators (PreLDL, PostLDL, MeanLDL, MinLDL, and MaxLDL) and applying robust statistical methods, this study seeks to provide scientific evidence for the early identification and intervention of postoperative complications in MMD patients.

## 2 Methods

### 2.1 Study design and patient selection

This retrospective, single-center study aimed to explore the relationship between dynamic changes in LDL levels during the perioperative period and the risk of postoperative cerebral infarction in patients with MMD, as well as to construct a risk prediction model. The study population included MMD patients who underwent surgical treatment at our hospital between 2015 and 2022. Inclusion criteria were: (1) a diagnosis of MMD confirmed by imaging (CTA, MRA, or DSA), with clear evidence of cerebral artery stenosis and ischemic manifestations; (2) complete perioperative lipid monitoring data; (3) at least 1 year of postoperative follow-up; (4) age ≥18 years. Exclusion criteria were: (1) severe liver or renal dysfunction or other systemic diseases; (2) a history of cerebral infarction prior to surgery; (3) insufficient duration of lipid-lowering medication to stabilize LDL levels; (4) missing data or loss to follow-up. Given the retrospective, single-center design, potential selection bias may exist, and these limitations will be further discussed in the discussion section. The study protocol was approved by the Ethics Committee of The Affiliated Hospital of Qingdao University (No.QYFY WZLL 29864) and adhered to the principles of the Declaration of Helsinki.

### 2.2 Data collection and LDL level assessment

Patient demographic data, medical history, laboratory test results, and imaging information were collected from the hospital’s electronic medical record system. Lipid profile data primarily included LDL levels, with measurement time points being preoperative (PreLDL), 24 h postoperative (PostLDL), and during the recovery period. Based on these data, the minimum (MinLDL), maximum (MaxLDL), and mean (MeanLDL) LDL values during the perioperative period were calculated. All blood samples were collected after overnight fasting and analyzed using standardized automatic biochemical analyzers in the central laboratory to ensure data consistency and accuracy. In addition to LDL levels, clinical characteristics including age, gender, smoking history, BMI, SBP, DBP, hypertension, hyperlipidemia, diabetes history, symptoms, Suzuki stage, Surgical type, modified Rankin Scale (mRS) scores at admission and one-year follow-up, and the presence of other cardiovascular diseases were also collected. Prior to data entry, all data were reviewed by two independent researchers to ensure completeness and accuracy, and appropriate preprocessing was performed. Missing data were imputed using multiple imputation methods to ensure the reliability and accuracy of the analysis results.

### 2.3 Statistical analysis and model construction

Data analysis was performed using SPSS 27.0 and R software (version 4.4.3). First, the Kolmogorov–Smirnov test was used to assess the normality of continuous variables. Normally distributed data are expressed as mean ± standard deviation, non-normally distributed data as median (interquartile range), and categorical variables as frequency and percentage. Comparisons between groups were made using t-tests or Mann–Whitney U tests for continuous variables and chi-square or Fisher’s exact tests for categorical variables. Subsequently, univariate analysis was used to identify variables significantly associated with postoperative cerebral infarction, and LASSO regression was employed for dimensionality reduction of the 18 candidate variables. The optimal penalty coefficient *λ* was determined using 10-fold cross-validation, and the “1-SE” criterion was applied to obtain the most parsimonious parameter combination. The retained LDL-related indicators included PreLDL, PostLDL, MeanLDL, MinLDL, and MaxLDL. In the model construction phase, multivariate logistic regression was used to analyze the selected variables, and the odds ratios (OR) and 95% confidence intervals (CI) were calculated to evaluate the independent associations between these variables and postoperative cerebral infarction. The model’s discriminative ability was assessed using ROC curves and AUC values, while calibration was evaluated using calibration curves and the Hosmer-Lemeshow test. Based on the multivariate analysis results, a visual nomogram was constructed to assist clinicians with individualized risk assessment. To further evaluate the clinical applicability of the model, DCA was used to assess the net benefit of the model at different risk thresholds. A *p*-value of <0.05 was considered statistically significant.

## 3 Results

### 3.1 Baseline characteristics and LDL level distribution

According to the inclusion and exclusion criteria, a total of 266 MMD patients were included in this study. Table 1 summarizes the baseline clinical data of these patients. The mean age at admission was 50.74 ± 4.36 years, with 166 male patients (62.41%). The preoperative LDL concentration was 3.48 ± 1.32 mmol/L, postoperative LDL concentration was 3.50 ± 1.43 mmol/L, the lowest recorded LDL concentration during hospitalization was 2.33 ± 1.07 mmol/L, and the highest LDL concentration was 4.70 ± 1.08 mmol/L. The average LDL concentration during hospitalization was 3.51 ± 1.19 mmol/L. Among the patients, 202 (75.9%) had ischemic-type Moyamoya disease, and 64 (24.1%) had hemorrhagic-type. Based on the Suzuki staging, there were 3 patients (1.13%) in stage I, 75 patients (28.20%) in stage II, 136 patients (51.13%) in stage III, 39 patients (14.66%) in stage IV, and 13 patients (4.88%) in stage V; no patients were in stage VI. All patients underwent revascularization surgery, with 149 (56.02%) receiving direct bypass surgery, 50 (18.80%) receiving indirect bypass surgery, and 67 (28.18%) receiving combined bypass surgery.

**Table 1:** Comparison between the clinical data of patients with MMD.

Characteristics	Postoperative cerebral infarction		*p* value
	Yes	No	
Total number (n)	64	202	
Demographic data			
Male	45 (16.9%)	121 (45.5%)	0.134
Age	51.31 ± 4.33	50.55 ± 4.37	0.226
Smoking	36 (13.5%)	112 (42.1%)	0.910
BMI (kg/m2)	23.27 ± 1.69	23.17 ± 1.69	0.685
SBP (mmHg)	127.73 ± 19.26	125.97 ± 21.27	0.555
DBP (mmHg)	78.02 ± 10.76	78.18 ± 12.21	0.922
Medical history			
Hypertension	32 (12%)	79 (29.7%)	0.124
Hyperlipemia	26 (9.8%)	61 (22.9%)	0.121
Diabetes	7 (2.6%)	26 (9.8%)	0.683
Coronary heart disease	2 (0.8%)	9 (3.4%)	0.910
Ischemic symptoms	54 (20.3%)	181 (68%)	0.256
mRS score(Admission)			0.277
1	9 (14.1%)	49 (24.3%)	
2	26 (40.6%)	68 (33.7%)	
3	25 (39.1%)	74 (36.6%)	
4	3 (4.7%)	9 (3.3%)	
5	1 (1.5%)	2 (0.1%)	
mRS score(1 year after surgery)			0.825
1	14 (21.9%)	53 (26%)	
2	25 (39.1%)	68 (33.7%)	
3	23 (35.9%)	73 (35.8%)	
4	2 (3.1%)	7 (3.4%)	
5	0 (0%)	1 (0.1%)	
Suzuki stage			0.935
Stage I	1 (0.4%)	2 (0.8%)	
Stage II	16 (6%)	59 (22.2%)	
Stage III	33 (12.4%)	103 (38.7%)	
Stage IV	10 (3.8%)	29 (10.9%)	
Stage V	4 (1.5%)	9 (3.4%)	
Stage VI	0 (0%)	0 (0%)	
Surgical type			0.586
Direct bypass	38 (14.3%)	111 (41.7%)	
Indirect bypass	13 (4.9%)	37 (13.9%)	
Combined bypass	13 (4.9%)	54 (20.3%)	
Laboratory values			
Pre-LDL (mmol/L)	3.76 ± 1.19	3.39 ± 1.35	0.049†
Post-LDL (mmol/L)	3.85 ± 1.39	3.39 ± 1.42	0.027†
Min-LDL (mmol/L)	2.76 ± 1.21	2.20 ± 0.99	0.002†
Max-LDL (mmol/L)	4.95 ± 0.98	4.62 ± 1.09	0.039†
Mean-LDL (mmol/L)	3.78 ± 1.21	3.42 ± 1.17	0.036†

When comparing the baseline characteristics between the postoperative cerebral infarction group and the non-infarction group, no significant differences were found in age, gender, smoking history, BMI, SBP, DBP, hypertension, hyperlipidemia, diabetes history, symptoms, Suzuki stage, Surgical type, modified Rankin Scale (mRS) scores at admission and one-year follow-up, and the presence of other cardiovascular diseases (*p* > 0.05), suggesting that these factors did not significantly affect the occurrence of postoperative cerebral infarction. Among the postoperative cerebral infarction group, 38 cases (59.38%) occurred after direct bypass surgery, 13 cases (26.31%) occurred after indirect bypass surgery, and 13 cases (26.31%) occurred after combined surgery. Preoperative LDL (3.76 ± 1.19 mmol/L vs. 3.39 ± 1.35 mmol/L), postoperative week 1 LDL (3.85 ± 1.39 mmol/L vs. 3.39 ± 1.43 mmol/L), minimum LDL (2.76 ± 1.21 mmol/L vs. 2.20 ± 0.98 mmol/L), maximum LDL (4.95 ± 0.98 mmol/L vs. 4.62 ± 1.09 mmol/L), and average LDL during hospitalization (3.78 ± 1.21 mmol/L vs. 3.42 ± 1.17 mmol/L) were all significantly higher in the infarction group compared to the non-infarction group (*p* < 0.05), indicating a close relationship between LDL fluctuations and the occurrence of postoperative cerebral infarction.

### 3.2 LASSO regression variable selection and risk model construction

**Figure 1:**
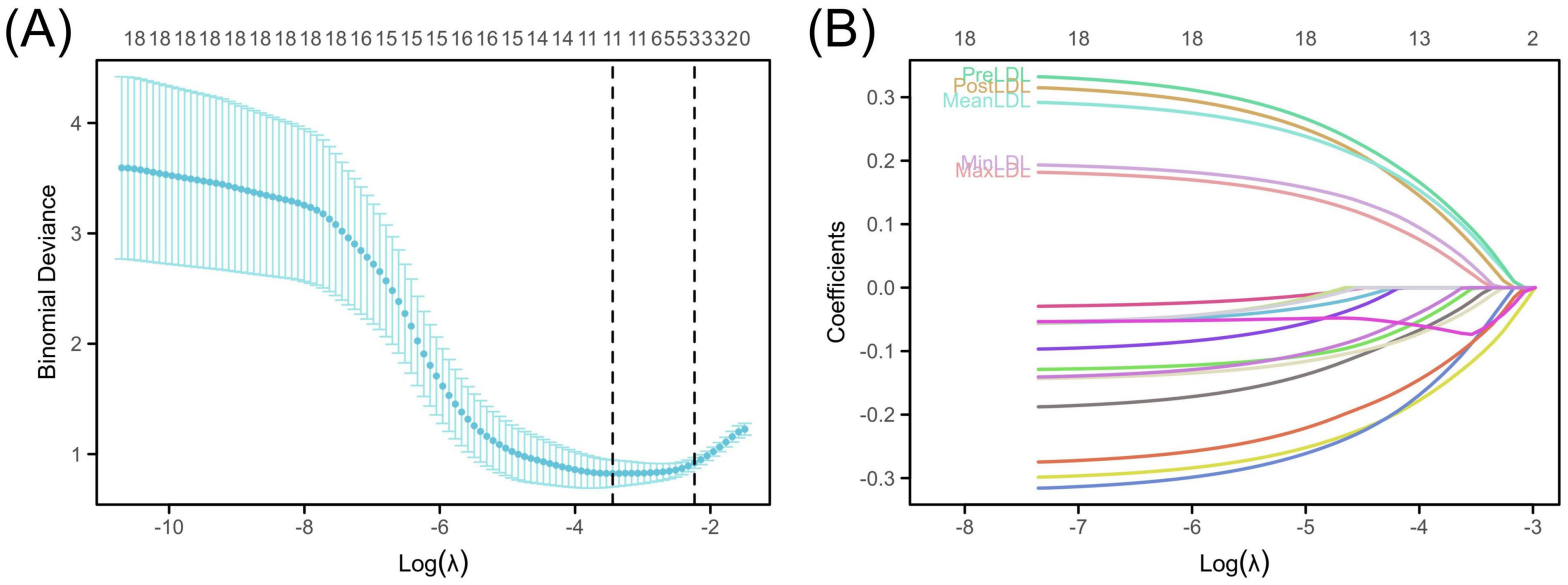
Construction of the LDL-based risk prediction model. **(A)** LASSO deviance profiles showing the optimal *λ* selected using the “1-SE” rule. **(B)** LASSO coefficient profiles illustrating the impact of LDL measures (PreLDL, PostLDL, MinLDL, MaxLDL, MeanLDL).

To select key LDL indicators closely related to postoperative cerebral infarction and construct a prediction model, the LASSO (Least Absolute Shrinkage and Selection Operator) regression method was used to reduce the dimensionality and select variables from 18 candidate variables. Figure 1A shows the trend of deviance changes at different penalty coefficients (*λ*): as λ increased, the coefficients of many less-contributing variables gradually shrank to zero, and the model deviance initially decreased and then increased. Based on the balance point between minimizing deviance and the “1-SE” criterion, five LDL-related indicators were retained, including PreLDL, PostLDL, MinLDL, MaxLDL, and MeanLDL (Figure 1B).

**Figure 2:**
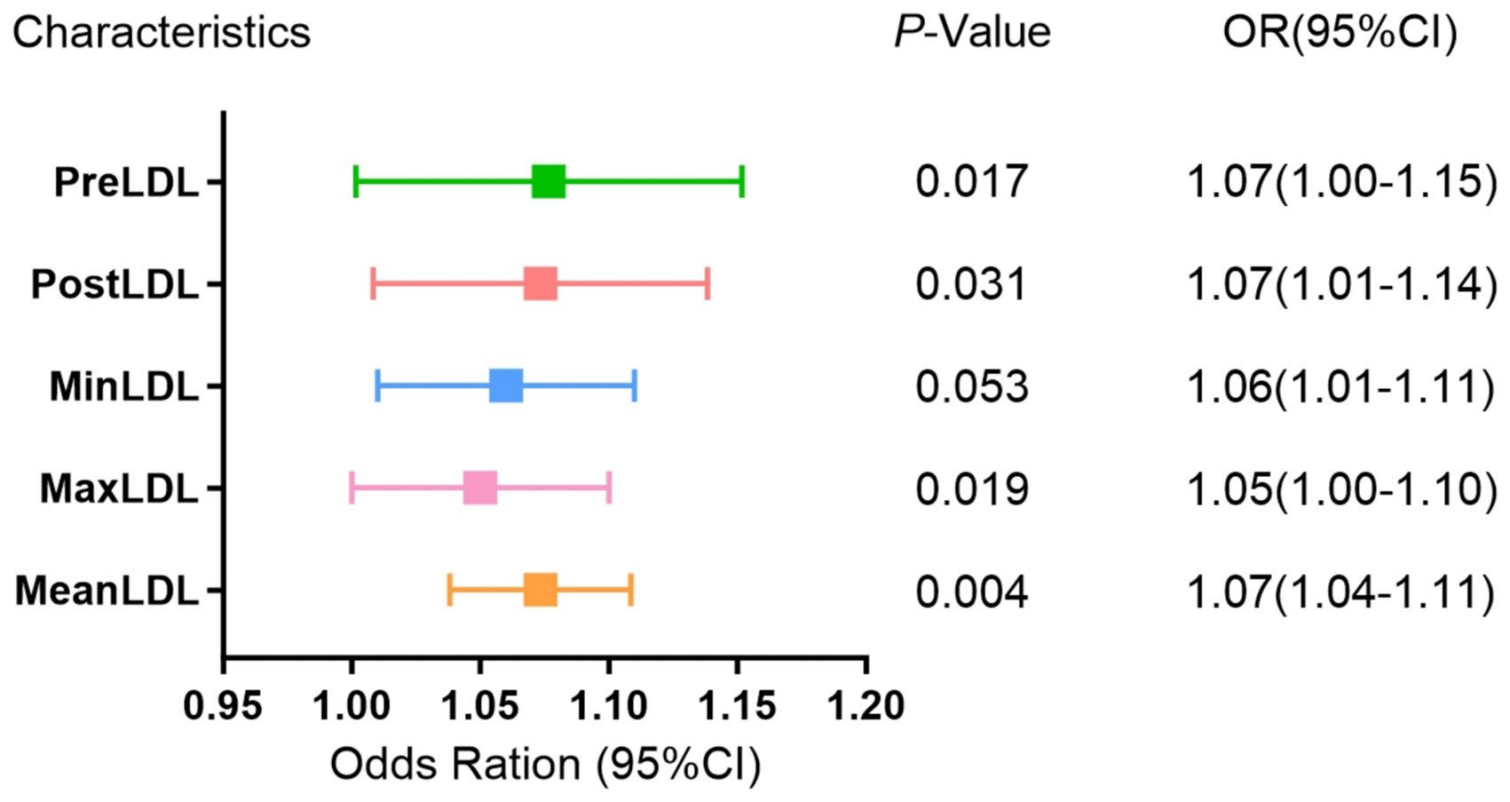
Multivariate binomial logistic regression analysis of LDL levels and postoperative cerebral infarction risk in MMD.

In the multivariate binary logistic regression model (Figure 2), all five indicators exhibited a positive association, suggesting that increases in these LDL indicators may raise the risk of postoperative cerebral infarction. Specifically, PreLDL (*p* = 0.017, OR = 1.07, 95% CI: 1.00–1.15) and PostLDL (*p* = 0.031, OR = 1.07, 95% CI: 1.01–1.14) showed statistical significance; MaxLDL (*p* = 0.019, OR = 1.05, 95% CI: 1.00–1.10) also had a significant predictive value for postoperative cerebral infarction. MeanLDL (*p* = 0.004, OR = 1.07, 95% CI: 1.04–1.11) had the highest coefficient, indicating its stronger contribution to the prediction model. MinLDL approached statistical significance (*p* = 0.053, OR = 1.06, 95% CI: 1.01–1.11), suggesting it may also have a potential impact on postoperative outcomes.

### 3.3 Risk prediction model construction and validation

To assess the predictive value of different LDL levels on postoperative cerebral infarction risk in MMD patients and construct a visual prediction model, this study systematically validated the model’s discriminative ability, calibration, and clinical applicability based on the core LDL-related indicators selected from LASSO and logistic regression (PreLDL, PostLDL, MinLDL, MaxLDL, MeanLDL).

**Figure 3:**
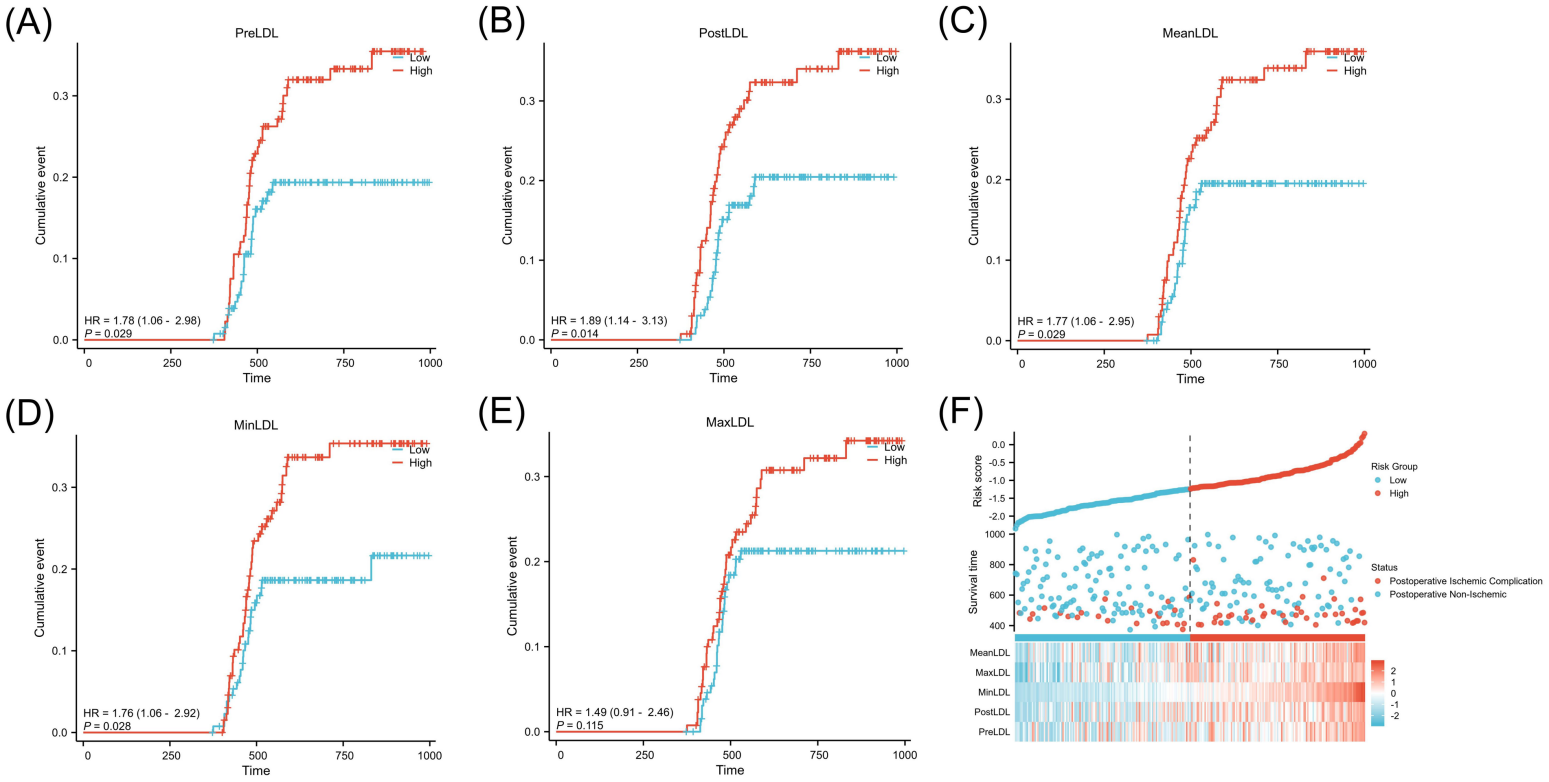
Kaplan–Meier analysis of postoperative cerebral infarction stratified by LDL levels in MMD patients.**(A)** PreLDL, **(B)** PostLDL, **(C)** MeanLDL, **(D)** MinLDL, **(E)** MaxLDL.**(F)** Risk stratification based on LDL, shown in a scatter plot and heatmap of clinical variables.

Figure 3 shows the cumulative incidence rates of adverse events (cerebral infarction) during follow-up for high and low LDL groups. After dividing patients into “high” and “low” groups based on the optimal cutoff value, Kaplan–Meier curves were plotted. The results indicated that the high LDL group exhibited a significantly higher cumulative incidence of postoperative cerebral infarction across all indicators (PreLDL, PostLDL, MeanLDL, MinLDL, MaxLDL) (all *p* < 0.01). For PreLDL, for example, the hazard ratio (HR) for the high PreLDL group was 1.78 (95% CI: 1.06–2.98, *p* = 0.029), suggesting that high PreLDL patients have a significantly increased risk of postoperative cerebral infarction. The other indicators also showed similar trends, with PostLDL showing the most significant association with postoperative outcomes (HR = 1.89, 95% CI: 1.14–3.13, *p* = 0.014). Figure 3F shows a heatmap of the major risk factors, highlighting that postoperative cerebral infarction events are more concentrated in the high LDL level population.

**Figure 4:**
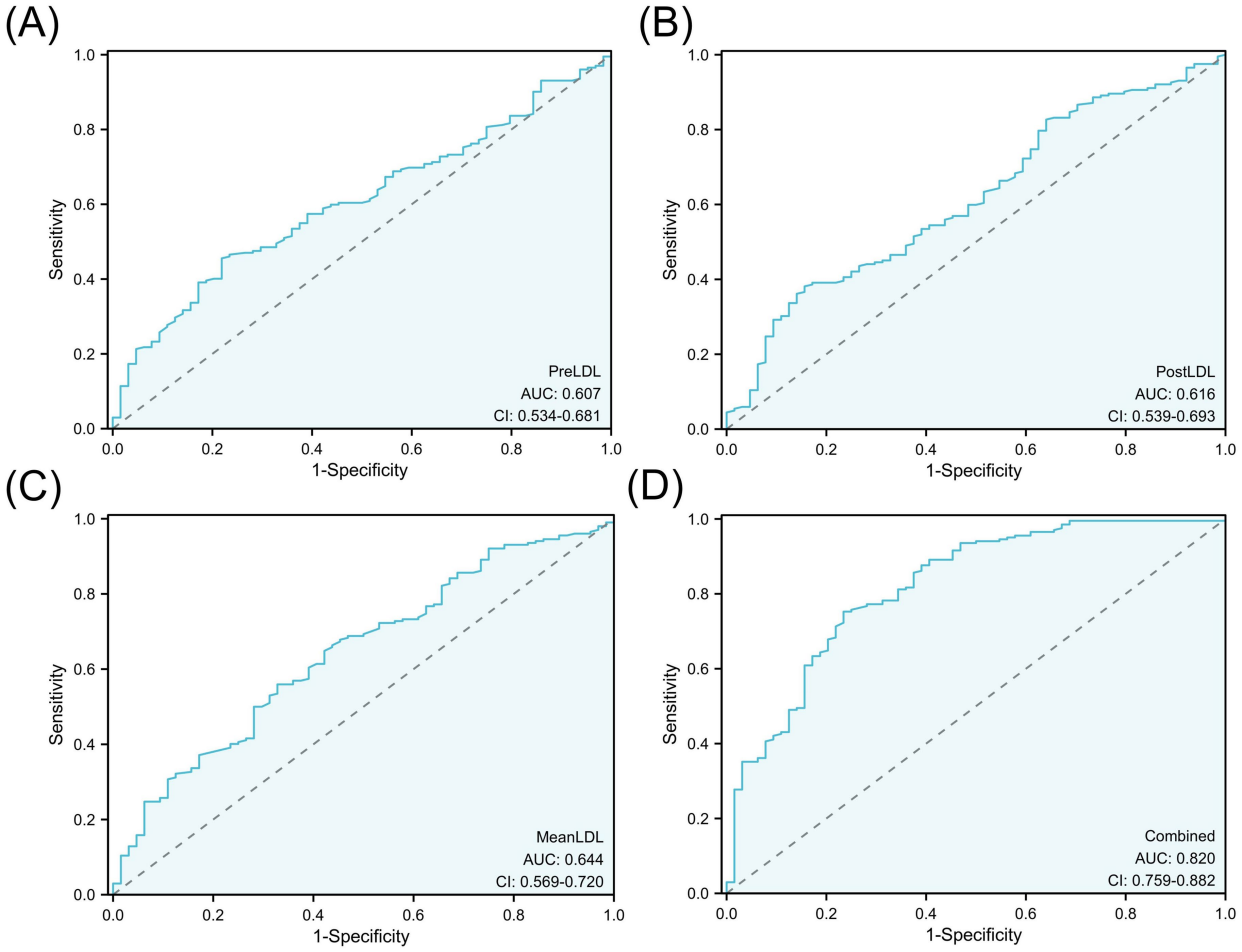
ROC curve analysis of LDL levels for predicting postoperative cerebral infarction.**(A)** PreLDL, **(B)** PostLDL, **(C)** MeanLDL, **(D)** Combined model. AUC values are shown.

**Figure 5:**
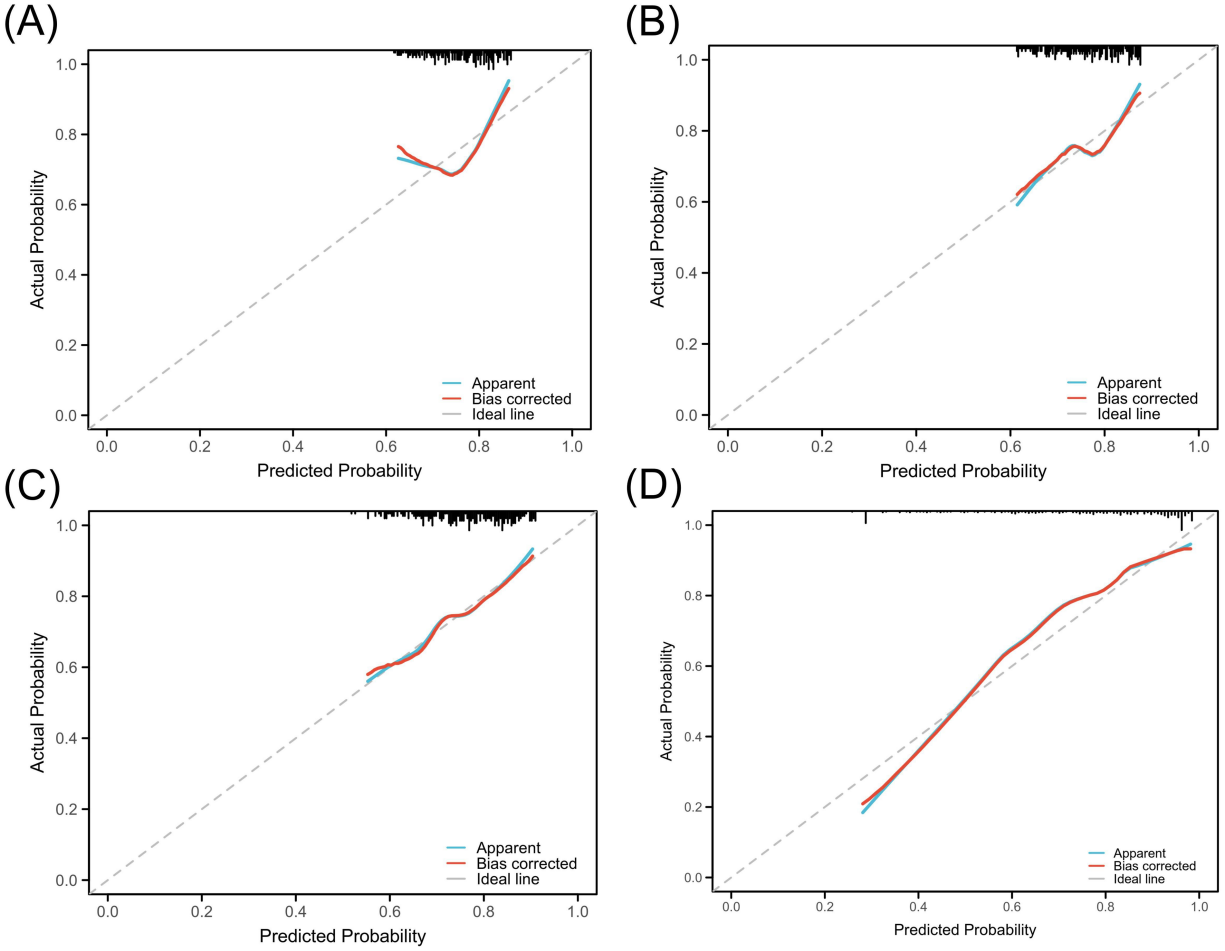
Calibration curves for the predictive model using LDL levels. **(A)** PreLDL, **(B)** PostLDL, **(C)** MeanLDL, **(D)** Combined. Calibration against the ideal line and bias-corrected curves.

Although MinLDL and MaxLDL were associated with some indicators of postoperative cerebral infarction in the analysis, their large fluctuations led to their exclusion in subsequent analyses to maintain model simplicity and robustness. The ROC curves were used to quantitatively evaluate the discriminative ability of individual LDL indicators and the combined model. The AUC values showed that PreLDL, PostLDL, and MeanLDL had AUCs of 0.607 (95% CI: 0.534–0.675), 0.616 (95% CI: 0.539–0.693), and 0.644 (95% CI: 0.560–0.720), respectively, indicating moderate discrimination (Figures 4A–C). After combining PreLDL, PostLDL, and MeanLDL with clinical features (such as age, gender, hyperlipidemia, smoking status) to construct the “Combined” model, the AUC significantly increased to 0.820 (95% CI: 0.750–0.892) (Figure 4D), demonstrating that the combined model outperforms individual indicators in both sensitivity and specificity (Table 2). To further validate the model’s goodness of fit, calibration curves were plotted. The calibration curves for each model showed minimal deviation from the ideal line, with the Combined model exhibiting higher fit in most risk probability ranges. The Hosmer-Lemeshow test revealed that the *p*-value for the Combined model was <0.01, indicating good calibration (Figure 5).

**Table 2:** The AUC, cut-off value, sensitivity, and specificity from the ROC curve (Based on the good prognosis group and the adverse prognosis group).

Characteristics	AUC	CI	Cut-off	Sensitivity (%)	Specificity (%)
Pre-LDL	0.607	0.534–0.681	3.035	0.45545	0.78125
Post-LDL	0.616	0.539–0.693	2.815	0.38119	0.84375
Mean-LDL	0.644	0.569–0.720	3.615	0.55941	0.67188
Combined	0.820	0.759–0.882	4.045	0.75248	0.76562

**Figure 6:**
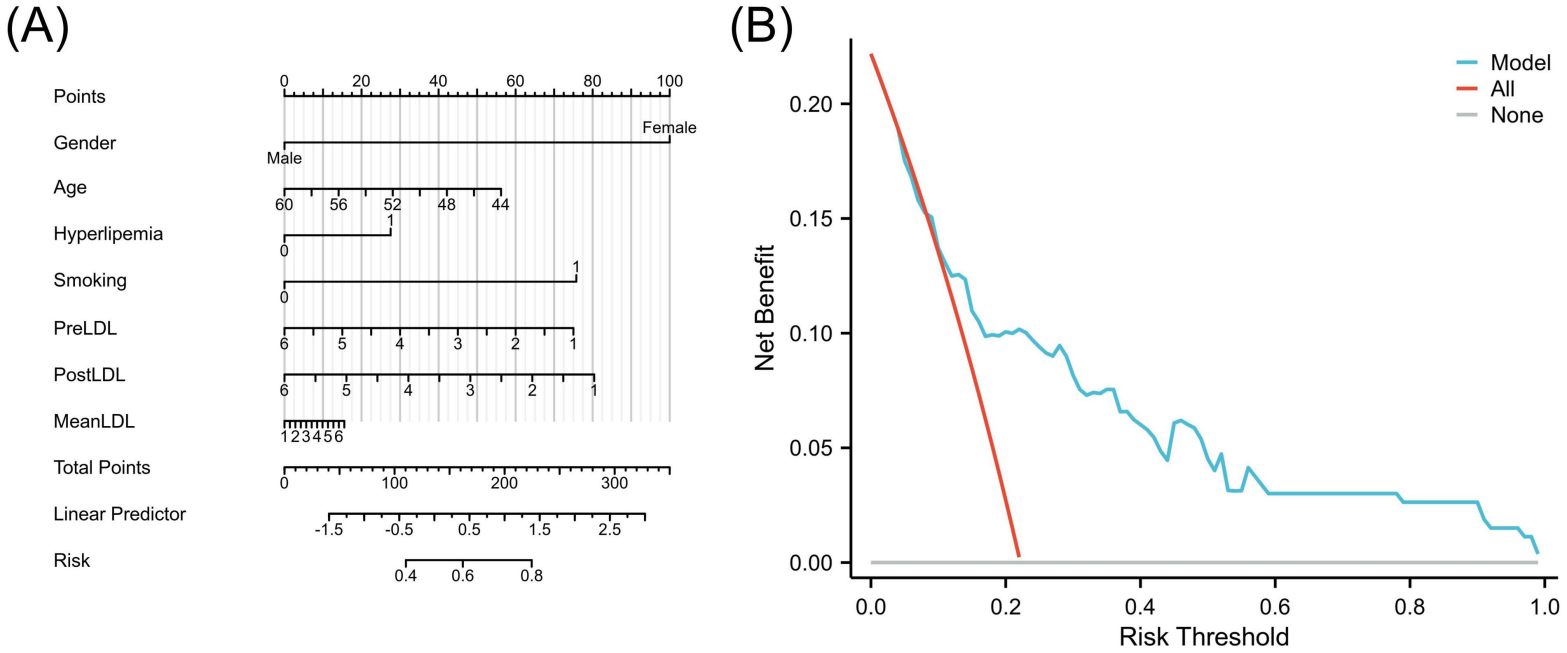
**(A)** Nomogram of the LDL-based risk prediction model for postoperative cerebral infarction. **(B)** Decision Curve Analysis (DCA) comparing the model with treating all or no patients.

The final risk prediction model considered gender, age, hyperlipidemia status, smoking history, and the three core LDL-related indicators (PreLDL, PostLDL, MeanLDL). For clinical use, a visual nomogram was generated using the R language rms package (Figure 6A), which allows clinicians to quickly assess individualized postoperative cerebral infarction risk based on the summed scores of each variable. Additionally, decision curve analysis (DCA) was performed to assess the clinical decision-making value of the model at different risk thresholds. The results showed that when the risk threshold ranged from 0.10 to 1.00, the Combined model provided significantly higher net benefit compared to the “all intervention” or “no intervention” strategies (Figure 6B). This suggests that the Combined model effectively distinguishes high-risk patients and reduces unnecessary over-intervention, thereby improving intervention efficiency.

## 4 Discussion

This study systematically evaluated the dynamic changes in LDL levels during the perioperative period and their association with the risk of postoperative cerebral infarction in patients with MMD. We also constructed a risk prediction model based on three key LDL indicators: PreLDL, PostLDL, and MeanLDL. The results showed that LDL levels at different time points were closely associated with postoperative cerebral infarction and exhibited significant predictive effects. While previous studies have primarily focused on single time-point LDL measurements, our study reveals the critical relationship between dynamic LDL changes and postoperative cerebral infarction risk by incorporating data from multiple time points. For instance, David et al. found an association between elevated LDL levels and cerebral infarction, but did not account for dynamic fluctuations (16). Our study addresses this gap by introducing LDL measurements at various time points. In the process of eliminating redundant variables, we not only excluded those with weak associations with prognosis but also carefully selected relevant clinical indicators related to LDL. For example, some clinical variables may be highly correlated with LDL levels, and these redundant variables could affect the model’s simplicity and reliability. Ultimately, we retained three core LDL indicators—PreLDL, PostLDL, and MeanLDL—which were significantly associated with the risk of postoperative cerebral infarction. These variables not only improved the predictive accuracy but also ensured the model’s practical applicability in clinical settings. Regarding the predictive effect of MeanLDL, studies suggest that LDL levels during the postoperative recovery period are particularly important. This indicator reflects the overall metabolic fluctuations during surgery and the recovery period, which may directly influence vascular repair and endothelial function. Thus, it holds unique clinical significance in predicting cerebral infarction.

From a pathophysiological perspective, LDL, as a key marker of lipid metabolism, plays an important role in endothelial damage, inflammation, and the progression of atherosclerosis (17–19). LDL promotes oxidative stress and the release of pro-inflammatory factors, exacerbating endothelial cell damage and dysfunction, a mechanism supported by multiple studies (20, 21). In MMD patients, progressive stenosis of the intracranial arteries due to both congenital and acquired factors, along with hemodynamic disturbances, make the vasculature more vulnerable to endothelial dysfunction and inflammatory stimuli caused by elevated LDL levels (22, 23). In this study, significant fluctuations in perioperative LDL levels may exacerbate the fragility of local blood vessels, increasing the risk of thrombosis and leading to cerebral infarction. By integrating preoperative and postoperative data, this study further emphasizes the importance of continuous monitoring of LDL dynamics. LDL not only reflects the patient’s lipid profile but also serves as an indicator of vascular health. Timely adjustments in LDL management could reduce the risk of perioperative complications. For example, prompt lipid-lowering therapy or adjustments may help prevent further vascular damage, thereby reducing the incidence of postoperative cerebral infarction (24–26).

By integrating clinical characteristics such as age, sex, hyperlipidemia history, and smoking history, we constructed a comprehensive prediction model that outperforms a model based solely on LDL measurements. Through multivariate analysis, the model achieved an AUC of 0.82 in the ROC curve and demonstrated strong concordance between predicted and actual incidence rates in calibration and Hosmer-Lemeshow tests. The advantage of the comprehensive model over single LDL indicators lies in its ability to combine multiple time-point LDL measurements with other clinical variables, providing a more comprehensive risk assessment. For example, a single LDL measurement may overlook changes in the patient’s metabolic state during the perioperative period, while the comprehensive model captures these changes over time, improving predictive accuracy. This finding suggests that relying on a single lipid measurement is insufficient to fully capture a patient’s risk. Integrating multi-timepoint LDL data with other clinical information can more accurately identify high-risk patients and provide a scientific basis for developing individualized treatment strategies. For example, clinicians can adjust the patient’s medication or surgical plan based on the model’s results, better meeting individual needs and achieving the goals of precision medicine (27).

Throughout the study, we considered the potential role of LDL in the pathogenesis of MMD and its relationship with the risk of cerebral infarction. LDL not only serves as a biomarker reflecting the overall metabolic state but may also directly participate in the local pathological process, influencing vascular repair and regeneration (28). Although this study controlled for several confounding factors, varying levels of statin therapy and other lipid-lowering interventions may still influence LDL levels and prognosis. Future studies should explore the role of statins and other lipid-lowering treatments in reducing the risk of cerebral infarction, particularly regarding how to adjust lipid-lowering therapy during the perioperative period to optimize patient outcomes (29). Furthermore, given the special role of elevated LDL in MMD patients, future research should further investigate the fine associations between LDL level changes and cerebral infarction risk in different subgroups. For example, multi-center prospective studies incorporating additional biochemical markers and imaging data could help us better understand the causal relationship between LDL and cerebral infarction, offering more precise clinical guidance for interventions. These research findings will further promote the individualized treatment of MMD patients, reducing postoperative complication rates.

In conclusion, this study, for the first time, constructed a comprehensive risk prediction model based on continuous dynamic monitoring data for MMD patients’ postoperative cerebral infarction risk, providing strong support for clinical risk assessment and intervention strategy development. The results emphasize the importance of LDL dynamic changes, suggesting that lipid management should not rely solely on a single lipid measurement but should focus on its time-series fluctuations to more accurately identify high-risk patients and facilitate intervention. Although this study has limitations, including a small sample size, a single-center design, and potential confounding intervention factors, the preliminary results in model construction and risk validation lay the foundation for future large-sample, multi-center research. Moving forward, integrating genetic, metabolic, and imaging data, as well as conducting randomized controlled trials to validate intervention effects, will contribute to advancing personalized precision medicine in MMD treatment, ultimately reducing postoperative complication rates and improving patient outcomes.

## 5 Conclusion

This study constructed a comprehensive risk prediction model based on dynamic monitoring of LDL levels during the perioperative period in patients with MMD. The model incorporates three core LDL indicators: PreLDL, PostLDL, and MeanLDL. The results show that the model has high discriminative ability in predicting the risk of postoperative cerebral infarction (AUC = 0.82) and has been validated through calibration and decision curve analyses, demonstrating good fit and clinical applicability. The findings emphasize the important role of dynamic monitoring of LDL levels in predicting the risk of postoperative cerebral infarction in MMD patients. This model provides a scientific basis for early identification of high-risk patients and the development of individualized intervention strategies.

## Data Availability

The original contributions presented in the study are included in the article/Supplementary material, further inquiries can be directed to the corresponding author.
